# Is there a rise of prevalence for Molar Incisor Hypomineralization? A meta-analysis of published data

**DOI:** 10.1186/s12903-023-03637-0

**Published:** 2024-01-25

**Authors:** Benjamin Sluka, Ulrike Held, Florian Wegehaupt, Klaus W. Neuhaus, Thomas Attin, Philipp Sahrmann

**Affiliations:** 1 Private Practice, Romanshorn, Switzerland; 2 Epidemiology, Biostatistics and Prevention Institute, University of Zurich, Zürich, Switzerland; 3 Clinic of Conservative and Preventive Dentistry, Center of Dental Medicine, University of Zurich, Zürich, Switzerland; 4 Department of General Pediatric and Adolescent Dentistry, University Center for Dental Medicine UZB, University of Basel, Basel, Switzerland; 5 Department of Periodontology, Endodontology and Cariology, University Center for Dental Medicine UZB, University of Basel, Basel, Switzerland

**Keywords:** Molar incisor hypomineralisation, Prevalence, Pedodontics

## Abstract

Since Molar Incisor Hypomineralization was first described as a pathologic entity, public perception often suggests a considerable rise in prevalence of the respective disease. Since there are still considerable doubts regarding the etiology and—accordingly—prevention of MIH and respective therapeutic approaches are difficult this question is of considerable clinical and public interest. Accordingly, a systematic literature search in accordance with the PRISMA guidelines for systematic reviews on Medline, Cochrane Database, EMBASE, LILACS, Web of Science, Google scholar, Scopus was performed to retrieve original articles reporting the prevalence of MIH as defined by the European Academy of Pediatric Dentistry (EAPD). From initially 2360 retrieved titles, 344 full texts were assessed for possible inclusion and finally 167 articles of mainly moderate to high quality and based on data of 46′613 individuals were included in the meta-analysis. All studies published before 2001 had to be excluded since it was not possible to align the findings with the EAPD classification. Studies varied considerably regarding cohort size (25 to 23′320, mean 1′235)) and age (5.6–19 y, mean 9.8 y). Over all studies, the weighted mean for the prevalence for MIH was 12.8% (95% CI 11.5%-14.1%) and no significant changes with respect to either publication year or birthyear were found. A sub-analysis of eleven studies reporting on the prevalence in different age groups, however, revealed strong evidence for an increasing prevalence between the years 1992 (3%) and 2013 (13%).

Therefore, based on data from cross-sectional studies a possible rise in prevalence of MIH remains unclear. Future prospective large-scale studies under standardized examination conditions with an emphasis on examiner calibration are needed to gain better understanding in the evolution of the prevalence of MIH.

**Supplementary Information:**

The online version contains supplementary material available at 10.1186/s12903-023-03637-0.

## Introduction

While hypomineralization in the deciduous and permanent dentition have been found in human teeth 100′000 years of age [1] and have been studied scientifically since over a hundred years [2] a new focus and special clinical interest has been set on this form of developmental tooth disorder in the industrialized and post-industrialized world when caries turned out to be a highly controllable scourge for both deciduous and permanent teeth [3]. In the late 90 s of the twentieth century hypomineralization in otherwise healthy first molars and incisors of the permanent dentition were described and – independently or as a consequence – observed more often [4]. This finding gave rise to concern, and in 2001 the respective manifestation was defined as a new dental disease and was then called the molar-incisor-hypomineralization (MIH). Already in 2003 the European Academy of Pediatric Dentistry stated the pediatric dentists’ awareness to this new dental disease’s symptoms, which was then defined as a hypomineralization of at least one first molar of the permanent dentition, which is often also accompanied by hypomineralization of the central and lateral incisors [5]. Since then, professional alertness towards MIH did not fade. Plenty of studies have been performed on etiology, epidemiology and different methods to treat MIH [6, 7]. While the exact etiology of MIH remains still unclear today’s understanding based on the available evidence is, that MIH has a multifactorial etiology: Genetic reasons with codings on multiple genes seem to constitute only one of umpteen predisposing factors [8]. Furthermore, MIH seems also to be associated with environmental risk factors, of which respiratory issues and infections, malnutrition, certain medication and vitamin D during childhood have been reported to have an effect [9–12].

Regarding the correct diagnosis for MIH, the special features and the typical localization of MIH lesions have to be carefully distinguished from other kinds of enamel hypomineralization (i.e., Turner teeth especially in case of incisors, initial carious lesions), hypomineralization (i.e., amelogenesis imperfecta) or other chemically induced disturbances during tooth development [13, 14]. The latter are often observed with a symmetrical pattern, while for the others careful clinical differential diagnosis (i.e., affection of all teeth in the case of amelogenesis imperfecta, localization of carious lesions in a dentition with active carious lesions) may lead to the definitive categorization.

For dental professionals, the treatment is often challenging because children affected by MIH are often less compliant due to frequently present tooth hypersensitivity [15, 16]. Furthermore, restorative treatment is technically challenging since it is still matter of discussion how deeply the altered tooth structure should be removed before restoration [17], and respective guidelines are subjected to constant changes [18, 19].

Other than for experts only, MIH has gained a huge repercussion in media [20]. Parents are concerned for their children’s dental wellbeing and studies show that MIH can in fact affect the parents’ quality of life [21].

Within the 20 years since the formulation of a generally accepted and well-established definition of MIH huge effort were taken to assess the prevalence of MIH. In fact, in the meantime, there is data based on a vast number of observational studies from literally nearly all parts of the world [22–24]. With symposia specifically held for MIH, media pushing the issue and health care providers advertising specific products for the treatment of MIH (https://www.aapd.org/education/meetings-calendar-aapd/international-symposium-on-molar-hypomineralisation-and-chalkyteeth/https://europe.gc.dental/sites/europe.gc.dental/files/products/downloads/equiaforteht/leaflet/LFL_Treatment_Solutions_for_MIH_de.pdf) there is the perception that MIH has become more and more prevalent since the specific problem had been addressed for the first time in 2001. To the best of the authors’ knowledge there has no systematical approach been made however to scientifically assess whether published data may substantiate the assumed fact of a rising prevalence. Accordingly, the focused question of this review was to assess, whether or not there is – based on the present data of epidemiologic studies – any robust indication for an increasing incidence of MIH. A special focus was set on the meta-analysis of studies reporting on subgroups of different age brackets and the question whether an increased prevalence in younger children was deductible.

## Materials and methods

The review was registered on PROSPERO (CRD42021243950) and performed following the guidelines for Preferred Research Items for Systematic Reviews and Meta-Analysis (PRISMA) [25].

Literature search included observational and case–control-studies (control groups were assessed if not adapted to special predisposing factors of the respective cases) which provided prevalence data for MIH according to the definition of the EAPD [5], Weerheijm [4] or if a definition was given that corresponded exactly to the latter.

The electronic search was performed on Medline, Cochrane Database, EMBASE, LILACS, Web of Science, Google scholar, Scopus according to a search strategy adapted to the specific database, combining different search items related to Molar Incisor Hypomineralization and prevalence and study design (see Additional file 1 Table 1).

The inclusion criteria comprised observational (cross-over or case control studies (only control group cohorts of the latter) reporting on the prevalence of MIH in populations which were not preselected (i.e., special exposition to environmental factors) and supposedly representative for the population.

MIH was accepted in terms of the MIH definition by EAPD 2003 [5] or Weerheijm [4] or DDE [26] with modifications which rendered an adaptation to the EAPD definition possible. It was planned to exclude all publications which would not clearly define hypomineralizations of the first molars which were not found to be due to dental fluorosis, enamel hypoplasia, or amelogenesis imperfecta.

No language restrictions were set.

Exclusion criteria encompassed studies assessing specific cohorts of populations which were not rated representative for the whole population, i.e., populations with special medical background and diseases, or cohorts exposed to special environmental factors, or studies addressing one of the sexes only. Likewise, studies which did not report on a clinical method of diagnosis of MIH (i.e., photos, scans, etc.) were excluded.

Duplicates from the electronic search were removed. In a first step titles and abstracts were screened for possible inclusion by two independent reviewers. Studies were kept in the screening process if at least one reviewer included it for full text assessment. Subsequently, full texts were assessed for possible inclusion. In order to avoid overestimation of cohorts that were assessed several times, only data of one study were considered. In this regard, the publication with the most comprehensive data was included to the review, or – as a secondary criterium when data was equally comprehensive, the most recent study was considered.

From studies accepted for inclusion and from reviews regarding the topic, reference lists were scrutinized for further studies potentially meeting the inclusion criteria, and potential hits were likewise considered for inclusion.

In a final step data from all full texts which finally fit to the inclusion criteria were extracted and recorded addressing the following items: Author/year, region of clinical examination, clinical assessment period, sampling environment (i.e., dental clinic, school room,…), study type (cross-sectional or case–control), MIH definition, equipment used for the clinical examination (i.e., air drying, headlight, etc.), cohort characteristics/population (age and gender proportions, provenience), cohort size, prevalence of MIH (and which teeth were reported), treatment needs, funding sources and data regarding the quality assessment of the studies (Table 1).

**Table 1:** Summary of the included studies. a) studies reporting prevalences at follow-up assessments for different age groups b) studies reporting general prevalence over the entire cohort

Author year	Examination year	MIH criteria	Examination environment	Study allocation	Region	Cohort size	Age range	Male proportion	MIH prevalence
**a) Studies reporting prevalences at follow-up appointments for different age groups**									
Abdelaziz 2022 [27]	2017	1	dental clinic	Geneve, Switzerland	Western Europe	23320	6–12	ng	6.6
Amend 2021 [28]	2015	1	classroom	Urban Hassonia, Germany	Western Europe	2103	6–12	52.2	13.5
Hong 2017 [29]	2015	1	ng	Suzhou China	East Asia	1145	7–12	53	4.45
Kukleva 2008 [30]	2006	1	classroom	Plovdiv, Bulgaria	Central Europe	2960	7–14	50	3.58
Oyedele 2015_b [31]	2011	2	classroom	Nigeria	Western Sub-Sahara	2107	12.57 ± 2.39	46.6	12.7
Quispe 2021 [32]	2019	1	classroom	Puno, Peru	Latin America & Caribbean	404	8.4 ± 0.9	44.1	19.8
Rai 2019 [33]	2018	1	classroom	Karnataka India	South Asia	1600	9–12	49.1	13.12
Thakur 2020 [34]	2017	1	classroom	Pradesh India	South Asia	2000	8–16	51.7	2.9
Verma 2022 [35]	2019	1	classroom	Lucknow, Indien	South Asia	5585	8–16	50.0	7.6
Yannam 2016 [36]	2012	1	classroom	Chennai India	South Asia	2864	8–12	52.3	9.7
Yi 2021 [37]	2015	1	classroom	Beijing China	East Asia	6523	12–15	49.5	10
**b) Studies reporting general prevalence over the entire cohort**									
Ahmad 2019 [38]	2013	1	classroom	Dubai gov schools	North Africa and Middle East	779	8.1 ± 0.8	33.9	7.59
Ahmadi 2012 [39]	2009	2	classroom	Zahedan, Iran	North Africa and Middle East	433	7–9	49.7	12.7
Al-Hammad 2018 [40]	2015	1	classroom	Riyadh, Saudi-Arabia	North Africa and Middle East	924	8–10 (9.1)	48.4	40.7
Allazzam 2014 [41]	2011	1	dental clinic	Jeddah, Saudi-Arabia	North Africa and Middle East	267	8–12, 9.4 ± 1.4	50.2	8.6
Americano 2017 [42]	2012	1	dental clinic	RiodJaneiro, Brazil	Tropical Latin America	98	9.26 ± 0.8	54.1	13.3
Arheiam 2021 [43]	2019	1	classroom	Benghazi, Libya	North Africa and Middle East	1047	8–11	47.5	15.5
Arslanagic 2020 [44]	2009	1	classroom	Sarajevo, Bosnia	Central Europe	444	6–9	47	11.5
Bahrololoomi 2017 [45]	2014	1	classroom	Yazd City	North Africa and Middle East	645	7–11	ng	23.9
Balmer 2015a [46]	2002	2	dental clinic	ng	Western Europe	25	11.3 ± 2.41	36	40
Balmer 2015b [47]	2002	2	dental clinic	Sydney AUS	High-income Asia Pacific	25	11.9 ± 2.31	28	44
Balmer 2012 [48]	2009	2	classroom	ng	Western Europe	3233	12	ng	15.9
Bhaskar 2014 [49]	2012	1	dental clinic	Udaipur India	South Asia	1173	8–13	54.3	9.46
Biondi 2011 [50]	2010	1	dental clinic	Buenos Aires Argentina	Southern Latin America	1098	11.3	ng	15.9
Biondi 2012 [51]	2010	1	dental clinic	Buenos Aires / Montevideo	Southern Latin America	975	11.6 ± 2.67	50	6.56
Bonzanini 2021 [52]	2019	1	classroom	Southern Brazil	Latin America and Caribbean 5	513	11.6 ± 1.9	45.2	19.7
Buchgrabner 2018 [53]	2010	1	dental clinic	Graz, Austria	Western Europe	1111	9.0 ± 1.2	49.2	7
Calderara 2005 [54]	2001	1	classroom	Lissone I	Western Europe	227	7.7	50.2	13.7
Da Costa Silva 2010 [55]	2008	1	classroom	Bothelos Brazil	Tropical Latin America	918	6–12	44.7	19.8
Da Costa Silva 2017 [56]	2012	1	classroom	Bothelos Brazil	Tropical Latin America	142	5.6	ng	16.19
Da Silva 2020 [57]	2016	1	dental clinic	Brazil (Rio de Janeiro)	Tropical Latin America	407	7–14, 10.1 ± 2.1	62.7	14.5
Da Silva J. 2015 [58]	2013	1	classroom	Belém Brazil	Tropical Latin America	260	10.22	56.9	8.84
Dantas-Neta 2016 [59]	2012	1	classroom	Teresina Brazil	Tropical Latin America	594	11–14, 12.45 ± 1.11	ng	18.4
Davenport 2019 [60]	2015	1	classroom	Milwaukee USA	High-income North America	375	7–12, 8.66	37.9	9.6
Dietrich 2003 [61]	2002	2	classroom	Dresden Germany	Western Europe	2408	10–17	ng	5.6
Ditto 2018 [62]	2017	1	classroom	Bahrain	North Africa and Middle East	760	7–9, 8.4	48.3	17.8
Dourado 2020 [63]	2016	1	classroom	Sao Raimundo Brazil	Tropical Latin America	246	8–14, 10.83 ± 1.93	53.7	46.6
Duarte 2021 [64]	2017	0	classroom	Paranoa, Brazil	Latin America and Caribbean 6	400	12 ± 1	42.8	18
Durmus 2013 [65]	2011	1	dental clinic	Marmar Turkey	North Africa and Middle East	228	7–14, 9.9 ± 1.7	54.4	24
Einollahi 2020 [66]	2019	1	classroom	Ardebil Iran	North Africa and Middle East	520	8–10	46.5	24
Elfrink 2012 [67]	2010	1	dental clinic	Rotterdamm	Western Europe	6161	6.2, 2.5SD	50.2	8.7
Elsoud 2019 [68]	2019	1	classroom	Suez-Canal-Region Egypt	North Africa and Middle East	1312	8–12	54.1	9.98
Emmatty 2020 [69]	2017	1	classroom	Kerala India	South Asia	5318	8–15	50.9	4.1
Farias 2021 [70]	2018	1	dental clinic	Medelin Columbia	Central Latin America	453	14.4, SD1.1	49.7	31
Farias L. 2021 [71]	2018	1	classroom	Campina Grande Brazil	Tropical Latin America	471	8–10	43.7	9.8
Fatturi 2020 [72]	2017	1	classroom	Curitiba Brazil	Tropical Latin America	731	8	51.2	12
Fernandes 2020 [73]	2019	1	classroom	Sao Joao do Rio Peixe Brazil	Tropical Latin America	610	6–12	53.9	9.8
Flexeder 2020 [74]	2017	1	ng	Munich, Germany	Western Europe	730	15	ng	13.8
Folayan 2018 [75]	2010	1	classroom	Southwest Nigeria	Western Sub-Saharan Africa	853	6–16	51.3	2.9
Fragelli 2021 [76]	2014	1	classroom	Araraquara Brazil	Tropical Latin America	467	8–12	45.8	19.7
Freitas-Fernandes 2021 [77]	2019	1	classroom	Campina Grande Brazil	Tropical Latin America	463	11–14, 12.1	36.7	10.8
Fteita 2006 [78]	2004	1	classroom	Beghazi Libya	North Africa and Middle East	378	7.8	50.3	2.9
Gambetta-Tessini 2018 [79]	2015	1	ng	Melbourne AUS	High-income Asia Pacific	327	6–12	52.9	14.7
Gambetta-Tessini 2019 [80]	2016	1	classroom	Talca Chile	Southern Latin America	577	6–12	48.2	15.8
García Margarit 2014 [81]	2009	1	classroom	Valencia	Western Europe	840	8	51	21.8
Georgieva-Dimitrova 2019 [82]	2018	1	dental clinic	Varna Bulgaria	Central Europe	1183	6–12	ng	6
Ghanim 2012 [83]	2010	1	classroom	Mosul Iraq	North Africa and Middle East	823	7–9, 8.1	57.2	18.6
Ghanim 2014 [84]	2012	1	classroom	Mosul Iraq	North Africa and Middle East	810	9–11	44.4	20.2
Glodwanska 2020 [85]	2018	1	classroom	Silasian, Pomeranian Poland	Central Europe	2354	6–12, 8.8, SD 1.89	50	9.32
Gorbatova 2019 [86]	2016	1	ng	North-West Russia	Eastern Europe 20	1233	12	47	2.1
Goswami 2019 [87]	2016	1	ng	Dheli India	South Asia	1026	6–12, 9.17, SD1.17	52	1.17
Groselj 2013 [88]	2005	2	dental clinic	Slovenia	Central Europe	478	9.1 ± 1.4	55.6	21.4
Gurrusquieta 2017 [89]	2014	1	classroom	Mexico City	Central Latin America	1156	8.4 ± 1.6	49.7	15.8
Gutierrez 2019 [90]	2018	1	ng	Naucalpan Mexico	Central Latin America	411	8–10	ng	40.4
Hamdan 2020 [91]	2015	1	classroom	Amman Jordan	North Africa and Middle East	1412	8–9	51.6	13.17
Hanan 2015 [92]	2014	1	classroom	Campina Grande Brazil	Tropical Latin America	2062	6–10	50	9.12
Hartsock 2020 [93]	2019	1	dental clinic	Milwaukee USA	High-income North America	104	5–32, 17.4	38.5	9.6
Hernandez 2018 [94]	2016	1	dental clinic	Barcelona Spain	Western Europe	705	6–15	48.8	7.94
Hertel 2017 [95]	2011	2	classroom	Dresden Germany	Western Europe	7051	7–9	49.9	5.6
Hoyte 2020 [96]	2016	1	classroom	Trinidad and Tobago	Latin America and Caribbean	532	11.9	43	1.3
Hussain 2018 [97]	2015	1	ng	Dubai	Andean Latin America	342	9.4 ± 1.2	37.1	27.2
Hussein 2015 [98]	2012	1	dental clinic	Malaysia	Southeast Asia	154	9.14 ± 1.682	43.5	16.9
Hysi 2016 [99]	2014	1	classroom	Tirana Albania	Central Europe	1575	8.84 ± 0.85	52.8	14
Irigoyen-Camacho 2020a [100]	2008	1	classroom	Mexico City 2008	Central Latin America	232	7.0(± 0.63)	50.4	20.3
Irigoyen-Camacho 2020b [100]	2017	1	classroom	Mexico City 2008	Central Latin America	317	7.1(± 0.62)	46.1	31.9
Jalevik 2001 [101]	1999	0	classroom	Kallered, Mölndal Sweden	Western Europe	516	8.3	51	18.4
Jalevik 2018a [102]	2012	0	dental clinic	Vestra Sweden	Western Europe	263	11	ng	17.1
Jalevik 2018b [102]	2012	0	dental clinic	Vestra Sweden	Western Europe	267	15	ng	11.2
Jalevik 2018c [102]	2012	0	dental clinic	Vestra Sweden	Western Europe	266	19	ng	8.3
Jankovic 2014 [103]	2006	1	classroom	Foca Bosnia Herzegovina	Central Europe	141	8	50.4	12.8
Jasulaityte 2007 [104]	2005	1	classroom	Kauna Lithuania	Eastern Europe 20	1277	7–9, 7.9	50.7	9.7
Jasulaityte 2008 [105]	2003	1	dentalbus	Kauna Lithuania	Western Europe	442	9	50.2	14.3
Jeremias 2013 [106]	2009	1	outdoor	Campina Grande Brazil	Tropical Latin America	1157	6–12, 8.84	46.2	12.3
Jurlina 2020 [107]	2017	1	classroom	Eastern Croatia	Central Europe	729	8.21 (± 0.12)	51.2	13
Kemoli 2008	2006	1	classroom	Matungula, Kangudno Kenia	Eastern Sub-Saharan Africa	3591	6–8	55.6	13.73
Kevrekidou 2015a [108]	2013	1	classroom	Greece	Western Europe	1179	8	ng	21.5
Kevrekidou 2015b [108]	2013	1	classroom	Greece	Western Europe	1156	14	ng	21
Kilinc 2019 [109]	2017	1	dental clinic	Izmir Turkey	North Africa and Middle East	1237	9–10	ng	11.5
Kim 2016 [110]	2013	1	ng	Melbourne AUS	High-income Asia Pacific	950	8–9, 8.4	58.2	7.1
Kirthiga 2015 [111]	2013	1	classroom	Davangere, Karnataka, India	South Asia	2000	11–16	58.7	8.9
Koruyucu 2018a [112]	2014	1	classroom	Istanbul, Turkey	North Africa and Middle East	719	8	ng	9.9
Koruyucu 2018b [112]	2014	1	classroom	Istanbul, Turkey	North Africa and Middle East	792	11	ng	18.2
Krishnan 2015 [113]	2014	1	classroom	Tamilnadu, India	South Asia	4989	8–14	43.3	7.7
Kühnisch 2014 [114]	2006	1	ng	Germany	Western Europe	639	10.2	52.3	14.7
Kühnisch 2018 [115]	2012	1	ng	Germany	Western Europe	1302	15.3	50	17.2
Kusku 2008 [116]	2007	1	dental clinic	Istanbul, Turkey	North Africa and Middle East	147	7–9	ng	14.9
Kuscu 2009 [117]	2007	1	classroom	Islands Turkey	North Africa and Middle East	153	7–10	ng	9.2
Li 2012 [118]	2009	1	ng	Wenzhou China	East Asia	988	6–11	ng	25.5
Llena 2020 [119]	2018	1	ng	Valencia Spain	Western Europe	278	12 ± 7 M	ng	23.02
Lopez 2014a [120]	2010	1	ng	Buenos Aires	Southern Latin America	1090	13.18 ± 2.55	ng	16.15
Lopez 2014b [120]	2010	1	ng	Montevideo	Southern Latin America	626	12.53 ± 2.68	ng	12.3
Lygidakis 2008 [121]	2004	1	ng	Athens, Greece	Western Europe	3518	5.5–12, 8.17 ± 1.38	ng	10.2
Mahima 2020 [122]	2014	1	ng	Pune India	South Asia	1400	8–11	50.7	7.4
Mahoney 2009 [123]	2008	2	classroom	Wainuiomata, New Zealand	High-income Asia Pacific	522	8.2 ± 1.2	46	14.9
Mahoney 2011 [124]	2008	2	classroom	Wellington New Zealand	High-income Asia Pacific	234	8.2 ± 1.1	50	18.8
Martinez-Gomez 2011 [125]	2009	1	dental clinic	Catalunya Spain	Western Europe	505	6–14	51.3	17.85
Martinovic 2017a [126]	2016	1	dental clinic	Kosovo	Central Europe	289	8	ng	10.7
Martinovic 2017b [126]	2016	1	dental clinic	Kosovo	Central Europe	423	10	ng	13.2
Mejia 2019 [127]	2012	1	classroom	Medellin, Colombia	Central Latin America	1075	9.3 ± 1.9	70.7	11.2
Menoncin 2019 [128]	2017	1	classroom	Curitiba brazil	Tropical Latin America	731	8	51.2	12
Mishra 2016 [129]	2013	1	ng	ng	South Asia	1369	8–12, 10.25 ± 1.25	ng	13.9
Mittal 2014 [130]	2010	1	classroom	Northern India	South Asia	1792	6–9, 7.66 ± 0.976	ng	6.31
Mittal 2016a [131]	2013	1	classroom	Gautam Budh Nagar, India	South Asia	1726	12–16	ng	13.21
Mittal 2016b [131]	2013	1	classroom	Nagpur India	South Asia	886	6–12	ng	7.11
Mulic 2017 [132]	2013	1	classroom	Kljuc, Bosnia Herzegovina	Central Europe	103	8–9	60.2	11.7
Munoz 2011 [133]	2008	1	dental clinic	La Frontera Chile	Southern Latin America	334	9.8 ± 1.9	50	16.8
Muratbegovic 2008 [134]	2004	1	classroom	Bosnia Herzegovina	Central Europe	560	12	ng	12.3
Murrieta-Pruneda 2016 [135]	2014	1	ng	Mexico	Central Latin America	433	8–12, 9.8 ± 1.3	54.3	13.9
Negre-Barber 2018 [136]	2013	1	dental clinic	Valencia Spain	Western Europe	414	8–9	51.2	24.2
Ng 2015 [137]	2011	1	dental clinic	Singapore	Southeast Asia	1083	7.7 ± 0.3	43.9	12.5
Nisii 2022 [138]	2020	0	classroom	Rome, Italy	Andean Latin America 3	346	8 ± 0.2	49.4	18.2
Nsour 2018 [139]	2015	1	ng	Amman Jordan	North Africa and Middle East	600	7–9	58	17.3
Ofi 2015 [140]	2014	1	ng	Al Najaf Iraq	North Africa and Middle East	532	7–9	54.3	25.8
Ordonez 2019 [141]	2016	1	classroom	Dubai	Andean Latin America	294	7–12	35.7	9.24
Orellana-Herrera 2020 [142]	2014	2	dental clinic	Talca City Chile	Southern Latin America	318	6	ng	19.8
Oyedele 2015_a [143]	2011	1	classroom	Nigeria	Western Sub-Saharan Africa	469	9.0 ± 0.9	54.4	17.7
Petrou 2014 [144]	2012	1	classroom	Germany	Western Europe	2395	8.1 ± 0.8	49.9	10.1
Pitiphat 2014a [145]	2012	1	classroom	Thailand	Southeast Asia	282	8.0 ± 0.5	47.5	27.7
Pitiphat 2014b [146]	2012	1	classroom	Thailand	Southeast Asia	484	6.5 ± 0.3	49.2	20
Portella 2019 [147]	2017	1	classroom	Curitiba brazil	Tropical Latin America	728	8	51.1	12.1
Poureslami 2018 [148]	2016	1	classroom	Kerman Iran	North Africa and Middle East	779	7–12	52.9	6.5
Preusser 2007 [149]	2004	1	classroom	Hessen Germany	Western Europe	1002	6–12	50.5	5.9
Rai 2018 [33]	2015	2	classroom	Muradnagar India	South Asia	992	7–9	53.6	21.4
Raposo 2019 [16]	2018	1	classroom	Brazil	Tropical Latin America	631	8	ng	16
Ray 2020 [150]	2018	1	ng	Odisha India	South Asia	1525	8–12	59	5.7
Reis 2021 [151]	2018	1	classroom	Minas Gerais, Brazil	Latin America and Caribbean 5	450	8	50.4	28.7
Rizk 2018 [152]	2017	1	classroom	Qassim Saudi Arabia	North Africa and Middle East	411	7–9	ng	25.1
Rodriguez-Rodriguet 2021 [153]	2020	1	classroom	Venezuela	Latin America and Caribbean 6	121	8.83 ± 1.61	42.3	25.6
Saber 2018 [154]	2015	1	dental clinic	Egypt	North Africa and Middle East	1001	8–12	49.9	2.3
Saitoh 2018 [155]	2015	1	dental clinic	Japan	High-income Asia Pacific	4496	7–9	49.3	19.8
Sakly 2018 [156]	2018	1	classroom	Tunisia	North Africa and Middle East	510	7–12	49.6	35.4
Salem 2016 [157]	2015	1	ng	ng	North Africa and Middle East	553	6–13	ng	18.4
Salih 2012 [158]	2009	1	ng	Baghdad Iraq	North Africa and Middle East	227	4–15	ng	6.61
Santos 2019 [159]	2015	1	classroom	Florianopolis Brazil	Tropical Latin America	1589	8.9 ± 0.8	42.4	9.5
Schmalfuss 2016 [160]	2011	1	ng	Northern Norway	Western Europe	794	16.6 ± 0.33	52.1	13.9
Shin 2010 [161]	2009	1	classroom	Südkorea	High-income Asia Pacific	1344	8–12	52.6	6
Shin 2017 [162]	2013	1	classroom	Südkorea	High-income Asia Pacific	1371	14–16	55.8	13.8
Shojaeepour 2020 [163]	2017	1	classroom	Kerman Iran	North Africa and Middle East	2507	10.5 ± 1.0	71.6	5.14
Shrestha 2014 [164]	2014	1	classroom	Nepal	South Asia	749	7–12	45.7	13.7
Sidaly 2017 [165]	2014	1	ng	Oslo	Western Europe	157	9.0 ± 0.8	49.7	25.4
Singh 2020 [166]	2018	1	classroom	Delhi India	South Asia	649	7–10	ng	15
Sönmez 2013 [167]	2006	1	classroom	Ankara Turky	North Africa and Middle East	4018	7–12	50.3	7.7
Sosa-Soto 2021 [168]	2018	1	classroom	Mexico	Central Latin America	613	8.6 ± 0.41	51.9	12.4
Subramaniam 2016 [169]	2013	1	classroom	Bengaluru City India	South Asia	2500	7–9	55.8	0.48
Tadikonda 2015 [170]	2014	1	classroom	Udupi India	South Asia	352	12.97 ± 1.2	51.1	27
Tagelsir 2020 [171]	2018	1	classroom	India	South Asia	337	9.1 ± 1.7	49.9	13
Tarannum 2021 [172]	2018	1	classroom	Pradesh, Indien	South Asia	2250	8–14	53.3	2.1
Temilola 2015a [173]	2012	1	haushold	ng	Western Sub-Saharan Africa	236	8–10	49.2	9.7
Temilola 2015b [174]	2013	1	haushold	ng	Western Sub-Saharan Africa	1169	7.2 ± 4.3	49.2	4
Tourino 2016 [175]	2014	1	outdoor	Florianopolis Brazil	Tropical Latin America	1181	8–9	49.3	20.4
Villanueva-Gutiérrez 2019a [176]	2018	1	ng	Mexico	Central Latin America	506	9.74 ± 1.36	50.6	42.4
Villanueva-Gutiérrez 2019b [177]	2016	1	ng	Mexico-City	Central Latin America	686	9.0 ± 1.4	46.8	35.4
Wuollet 2016 [178]	2013	1	dental clinic	Finland	Western Europe	287	10.4 ± 1.3	55.4	11.5
Wuollet 2018 [179]	2004	1	dental clinic	Finland	Western Europe	636	10.5 ± 1.4	51.7	18.1
Zakirulla 2018 [180]	2015	1	classroom	Saudi Arabia	North Africa and Middle East	596	8.5	100	21.3
Zawaideh 2011 [181]	2009	1	classroom	Jordan	North Africa and Middle East	3241	8.4 ± 0.7	52.5	17.6

Legend Classification: 1 – MIH classification according to the European Academy of Pediatric Dentistry (EAPD) or Weerheijm 2003 and 2 – according to the DDE-classification

### Quality assessment

With the aim to assess the risk of potential bias of the individual studies, a modified GRADE scale for risk assessment was used, involving the parameters representativeness of the cohort, sample size calculation, calibration of the examiners and case definition. If no high risk of bias for any of these parameters were detected, the study was rated as high quality, with one parameter with a high risk of bias the study was rated moderate and with more than one parameter with high risk of bias the study was rated as low quality.

### Statistical methods

The outcome of interest was the prevalence of MIH reported across studies. The summary estimate for MIH across studies was obtained with a random effects meta-analysis model, based on restricted maximum likelihood estimation. For each meta-analysis, the estimated heterogeneity variance parameter τ^2^ was reported together with the measure of heterogeneity I^2^. In graphical representations, the prevalences reported across studies were shown in relation to assessment period and birth period.

In order to show forest plots, subgroups of studies including different birth cohorts were displayed. Further, there were some studies that reported an age-dependent prevalence of MIH, with different follow-up times across studies. The reported prevalences are shown in a visualization, indicating which prevalence resulted from the individual cohort. In this context, a generalized linear regression model was fitted in order to evaluate in an exploratory approach, whether there was evidence for an increasing prevalence over time. For this, the repeated measurements across studies were addressed with a random intercept, but the size of the included studies was not accounted for in a meta-analysis framework. Statistical calculations were performed with the programming language R version 4.1.1 (2021–08-10) in a fully scripted approach using dynamic reporting.

## Results

The electronic search revealed 2′369 titles and abstracts. Of these, 344 full-texts were considered for possible inclusion. After removing studies reporting on the same cohort, studies which did not provide suitable data presentation for the prevalence of MIH as defined in the method chapter and studies which assessed cohorts obviously non-representative for the general population, data of 167 distinct studies were finally included into the meta-analysis.

None of the publications before 2001 were found to allow for a proper definition of hypomineralizations of the first molars, or rather a clear delimitation from dental fluorosis, enamel hypoplasia, or amelogenesis imperfecta. Accordingly, all these studies were excluded from the meta-analysis.

**Fig. 1:**
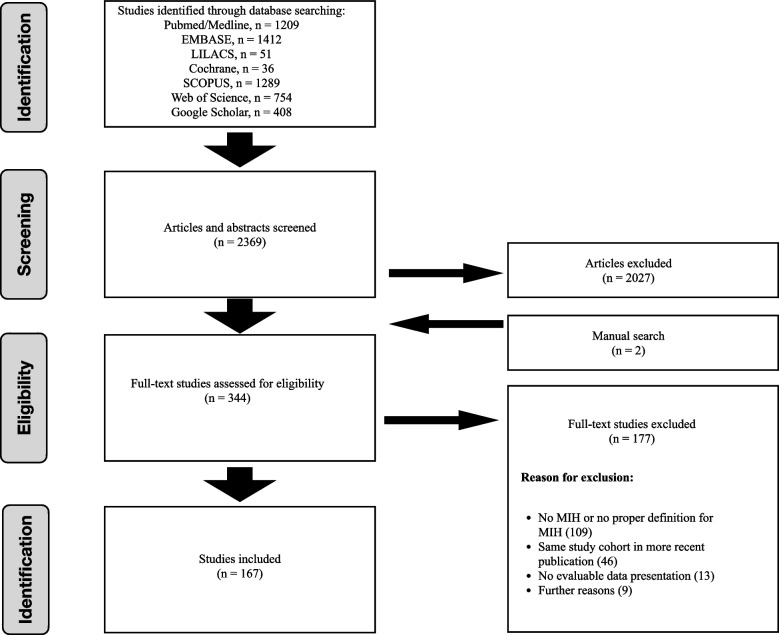
PRISMA flow diagram for the literature search

The entire study selection process is illustrated in Fig. 1.

### Quality assessment

While most of the studies included to the meta-analysis were of medium quality (148), the studies covered the whole range from high (58) over moderate (70) to low (31) quality. The most frequent reason for limited quality was missing sample size calculation (199) followed by missing inter-examiner reliability (134) (Additional file 1 Table 2).

Studies included to the review were published after 2001 but several examinations had taken place considerably before. Data from 46′613 individuals had been considered in the present review. The assessed cohorts in the different studies varied from 25 to 23′320 children with a mean cohort size of 1′235 infants across studies. The assessed population varied in age from 5.6 to 19 years of age and were at a weighted mean age of 9.8 years old. The weighed mean proportion of boys, where reported, was 51% across studies, ranging from 28 to 100% while in 42 of the included studies, no information on children’s sex was reported.

**Fig. 2:**
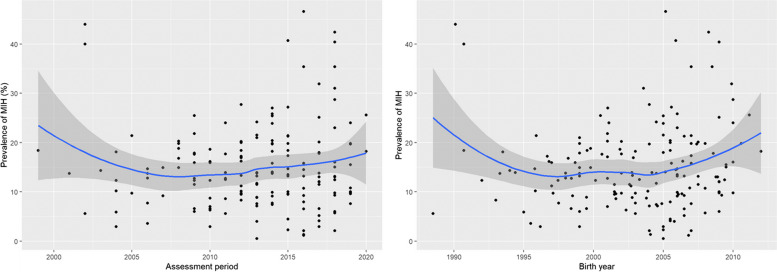
**a** and **b** Prevalences of MIH in the included studies. **A** Reported prevalences of MIH related to year of publication and **B**) related to birthyear of the respective cohort. Each black dot indicates the prevalence reported in an individual study. The blue line shows the loess-smoothed prevalence of all studies related to publication year or birth year, while the grey area indicates 95% confidence interval

Figure 2a indicates the prevalences of MIH related to the year of publication, while Fig. 2b shows the prevalences related to the birthyears of the study participants, by subtracting the cohort’s mean age from the year of examination. The prevalences were loess-smoothed, and the curve is shown with 95% confidence intervals.

Across all studies, the prevalence of MIH ranged between 0.48% and 46.6% resulting in a summary estimate across studies based on a random-effects meta-analysis model of 12.8% (95% CI 11.5%-14.1%), with τ^2^ being 0.56, and I^2^ being equal to 98.5%. To address the question whether a different classification scheme would affect the estimation of the prevalence, the classification type was added as a moderator in a random effects model. The corresponding *p*-value was 0.17, indicating that there was no evidence for a moderating effect of the classification scheme.

**Fig. 3:**
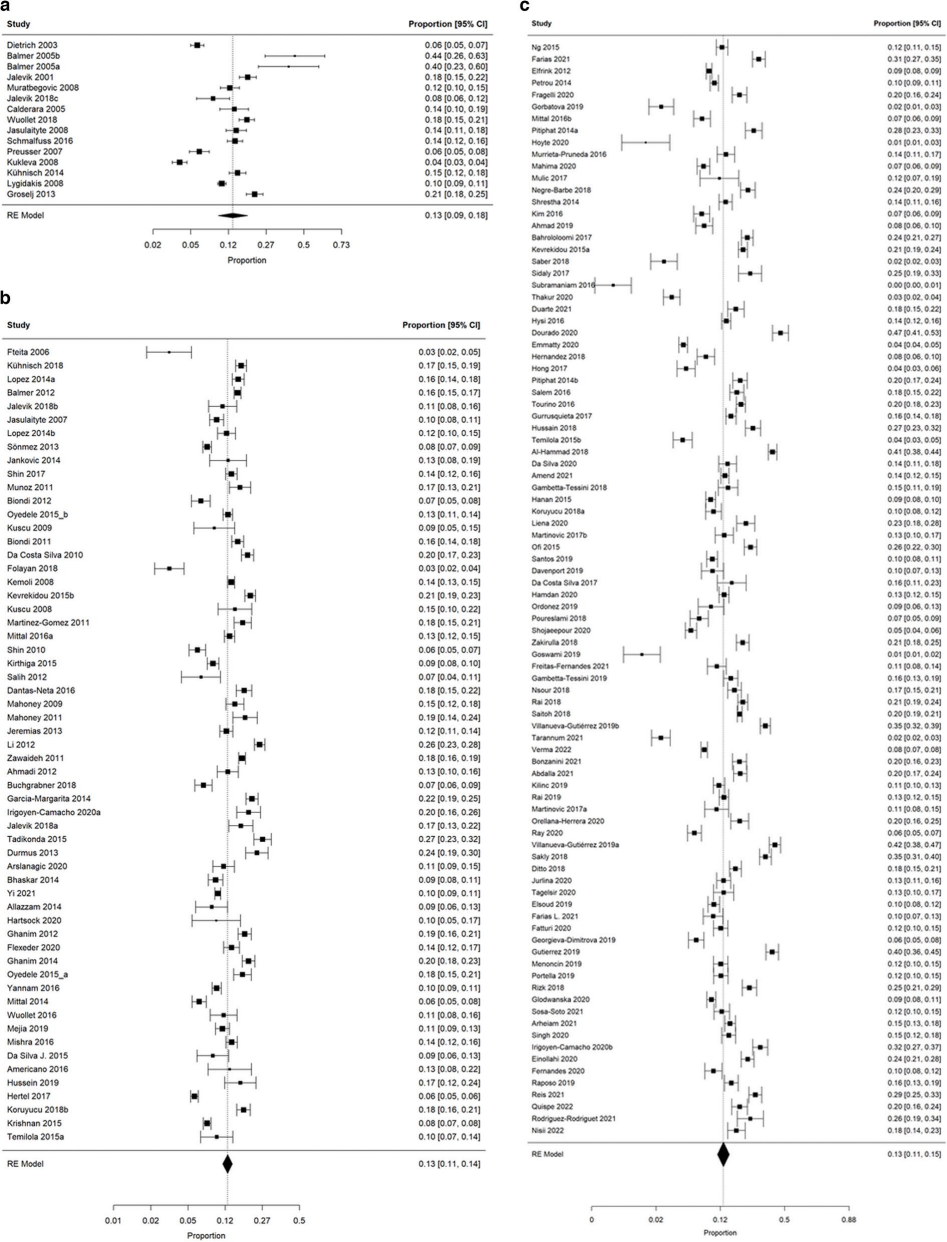
**a** Prevalences of MIH in studies reporting on populations with a mean birth year before 1997 The column on the left indicates first author and year of publication. The position of the boxes indicate the mean value for the prevalence of MIH while the size of the boxes indicates their weight in the meta-analysis. Whiskers depict the confidence interval (CI). Means and CI are given in the column on the right. The rhombus indicates the mean (position) and the CI (horizontal extention) of the overall summary estimate. RE indicates a random effects model for meta-analysis. The heterogeneity variance τ^2^ was estimated to be 0.56, I^2^ was estimated to be 97.7%. **b** Prevalences of MIH in studies reporting on populations with a mean birthyear from 1997 to 2003. The column on the left indicates first author and year of publication. The position of the boxes indicate the mean value for the prevalence of MIH while the size of the boxes indicates their weight in the meta-analysis. Whiskers depict the confidence interval (CI). Means and CI are given in the column on the right. The rhombus indicates the mean (position) and the CI (horizontal extention) of the overall summary estimate. RE indicates a random effects model for meta-analysis. The heterogeneity variance τ^2^ was estimated to be 0.25, I^2^ was estimated to be 96.8%. **c** Prevalences of MIH in studies reporting on populations with a mean birthyear after 2003. The column on the left indicates first author and year of publication. The position of the boxes indicate the mean value for the prevalence of MIH while the size of the boxes indicates their weight in the meta-analysis. Whiskers depict the confidence interval (CI). Means and CI are given in the column on the right. The rhombus indicates the mean (position) and the CI (horizontal extention) of the overall summary estimate. RE indicates a random effects model for meta-analysis. The heterogeneity variance τ^2^ was estimated to be 0.79, I^2^ was estimated to be 98.8%

To show the prevalence of MIH in forest plots, subgroups of birth year ranges were defined with the cut-off values for the birth years being A) before 1997, B) between 1997–2003 and C) from 2003 on (Fig. 3a-c). With overall estimates of the mean prevalence and 95% confidence intervals of 13% (9% to 18%), 13% (11% to 14%), and 13% (11% to 15%), respectively, there was no evidence for a difference between the subgroups of birth year categories.

Prevalence across studies with repeated follow-up assessments.

**Fig. 4:**
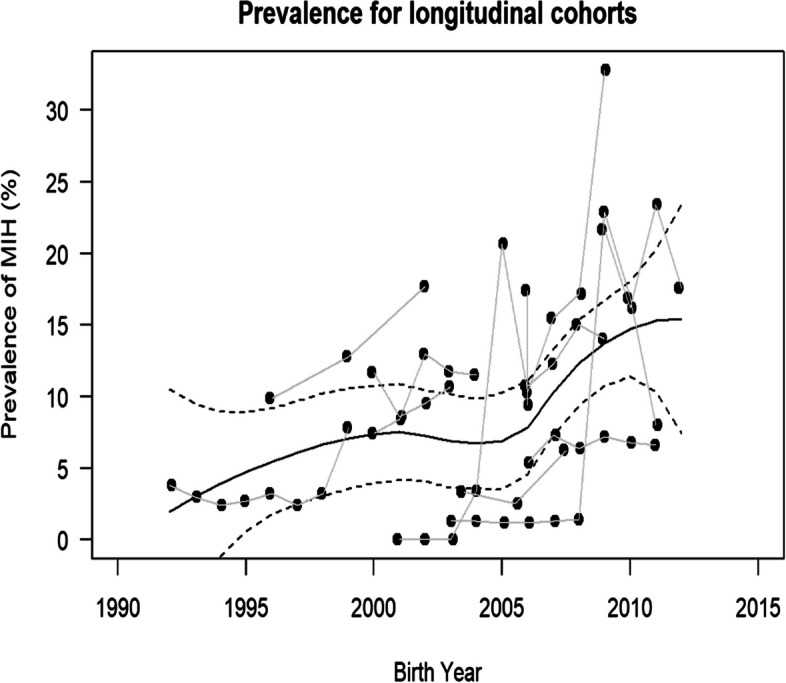
Prevalences of MIH in studies reporting separately on the prevalence of MIH for different ages. Each black dot indicates the prevalence of an individual study. Grey lines connect data from the same study population. The continuous black line shows the loess-smoothed prevalence across all study cohorts, the dashed lines indicate 95% confidence limits [27–30, 32–37, 143]

The analysis on the data of studies which reported repeatedly prevalences in groups at different ages was based on 11 studies (see Table 1a [27–30, 32–37, 143]). The respective data is based on a total of 50`611 (range 404–23320) patients with an aged from 6 to 15y from Western Europe and Asia. In all studies, MIH was diagnosed based on ethe EAPD criteria or according to Weerheijm. Regarding the proportion of males and females there was no relevant deviation. Generally, MIH prevalence ranged from 2.9 to 19.3 in the respective studies. It shows a continuous rise in prevalence for MIH from the earliest examination in 1992 to the most recent available in 2011 (Fig. 4). The corresponding p-value for a linear time trend showed strong evidence for an increasing prevalence. Based on the merged data on the respective studies the prevalence of MIH show a rise from 6 to 14% from the years 2000 to 2010. A sub-analysis of the prevalence over time shows considerable differences for the prevalence of MIH between the regions previously described by Schwendicke et al. [22]. While for most areas there are few studies and they are therefore not eligible for a sound analysis, the areas “North African and Middle East” and “Central Latin America” show a rise in prevalence while the areas “Tropical Latin America” and “High-income Asia Pacific” show a respective decline (see Additional file 1 Fig. 1).

## Discussion

Molar incisor hypomineralization has become an important topic in pediatric dentistry within the last 20 years [19, 182]. Since the perceived prevalence is continuously rising and the impact for affected children and their families are considerable [21] plenty of epidemiologic studies have been performed all over the world [22], but so far, no systematic approach has been investigating whether there is actually any indication for any rise in prevalence from scientific literature.

Merging data on weighted means of prevalence for MIH failed in either of the performed calculations to indicate a rise over time: Neither for the raw data presented in 167 studies nor after adjusting the data to the actual age of the populations in which the studies had been performed revealed an enhanced prevalence. Ideally, however, the question whether there is a raise in the prevalence of MIH would be answered by studies assessing children of the same age group after a considerable time span of several years under standardized and well-calibrated conditions. Therefore, in a separate analysis on data from studies with age-specific prevalences for the assessed children and adolescents was performed. The results draw a different conclusion: Based on this specific data from 11 high quality studies (see Supplemental Table 2), in fact, a considerable rise based on studies from Europe, Africa, Asia, South America and Oceania in the prevalence of MIH becomes evident. Important to highlight, however, that this result is not based on a meta-analysis model due to the complexity of the data structure with multiple reportings across single studies. For this reason, the results will need confirmation in prospective studies.

With an average of 12.8% the mean value from the studies considered for the present meta-analysis are slightly lower than those of two recent reviews with 13.1% and 14.2% [22, 183]. Nevertheless, the results are in the same range, and small differences may be attributed to the later publication date or recent publications which have not been considered in the previous reviews. Given that both, age distribution and proportion of males and females did not differ considerably to the previous reviews, renders the latter options for a potential bias rather unlikely and seems to confirm the relevance of more recent data.

The results of the present systematic review should however be considered with care: Many studies and reviews estimating the prevalence of MIH have been highlighting the limits of the individual reports (due to a considerable bias level as assessed also for the present study): The cohorts themselves might not always exactly depict the population in which the examinations were performed, the definition of MIH might slightly vary while data on the inter-examinators agreement have not always been reported. Furthermore, sample size calculations, a measure which aims for avoiding statistical type II errors, were not performed in all of the studies included into the present review (see Supplemental Table 2, quality assessment) [184]. Then it is important to highlight, that data on prevalence show a vast range from 0.48% and 46.6% within the different studies [185], a strong indicator for the fact that merged data is based on a highly heterogeneous set of data. Generally, merged analysis of data from cohort studies of all different areas of the world bear several immanent risks like different operators and settings, and published changes for the prevalence of MIH in distinct populations or single nations might remain unperceived [54, 138].

On the other hand, the results of this review are based on a comparatively high number of studies: With 167 studies on data of a total of 46′613 patients the results are based on a quite solid base and stands out from most comparative studies in dentistry.

Hypomineralization on first molars of the permanent dentition is not a new finding for sure. Several studies examining prehistoric dentitions show enamel demineralization with the typical features of MIH-affected teeth, namely an increasing mineralization from the enamel-dentine junction towards the tooth surface [186–188]. Furthermore, there are plenty of examinations which were performed in the late last century. Though they assess hypomineralizations on a standardized and highly scientific level, a good deal of the respective data could not be used in the present meta-analysis due to the fact that the addressed hypomineralizations had not been specified exactly before 2001 [4], and MIH as an entity has not been defined before 2003 [5]. Accordingly, the greatest part of 59 studies published from 1930 to 2002 had to be excluded, since a valid adaptation of the findings to the definition of MIH was not possible in these studies: Mostly, either the exact location on a permanent molar or a sufficient demarcation towards defects of similar appearance but of different origin like enamel altered by fluorosis, hypoplasia, Turner teeth or discoloration due to tetracyclines [13, 14] was not possible. However, as a lucky matter of fact several examinations which had been performed before 2001 and the definition of MIH were published considerably later, then considering the definition of MIH which had been published recently before. Luckily, the new definition of MIH was quickly spread and adapted on then-published studies, what helped to find comparable data and, therefore, considerable evidence.

The fact that the prevalence of MIH might indeed rise by time is an important puzzle piece in MIH research since quantification of global and regional burden of MIH and the awareness of respective trends and changes in the prevalence is highly relevant for decision makers in the healthcare sector and health care providers [22].

Data on the prevalence of MIH merged in the meta-analyses showed a considerable range. The respective heterogeneity might be to many different reasons: While there might be different sources of bias like different levels of specificity and sensitivity of the operators, partially attributable to the examination settings in which they performed their examinations (see Table 1), the simple fact that all over the world different ethnicities were assessed [22] over a considerable time frame will have contributed to variations in the prevalence data.

With a range from 5.6 to 19 years, there was a considerably large heterogeneity for the age of the examined patients. While some studies assessed patient cohorts at a defined age, others involved children of mixed ages, while other studies assessed the prevalence of MIH in several defined age groups in follow-up appointments (Table 1a). Prevalence of MIH, however, has been reported be age-dependent with a higher proportion in children younger than 10 years of age [183]. In order to standardize examinations and respective results, optimal time points (5y for hypomineralised second primary molars and 8y for MIH) have been suggested [189]. Regarding the inclusion criteria is should be highlighted, that the decision to include data from both, cross-sectional studies and from control cohorts of case–control studies generally bear a higher risk of Berkson’s bias, the latter arising due to testing a subpopulation rather than the general population [190]. This potential risk has been largely double-checked in the present study since the selection bias was assessed in the quality assessment. Thereby however, no substantial inferior quality for the controls cohorts form case–control studies could be determined.

The present review merges data from studies of different quality. The high proportion of low-rated studies might be seen as a limitation. However, low quality was already rated if more than on parameter was considered to have a high risk for a bias, thereby not rendering the study data unreliable, but highlighting the possible source of bias.

## Conclusion

Merged data from cross-sectional studies do not indicate a rise in worldwide’s prevalence of MIH. However, studies on age-specifical assessment show a rise from 6 to 14% in the years from 2000 to 2010 for the prevalence of MIH.

Further age-specific re-analysis of existing data on one side and future studies, which assess age-dependent prevalence of MIH on a high-quality level might unearth more knowledge about the dynamic of MIH prevalence than further unspecific reports on just mean prevalences.

### Supplementary Information

**Additional file 1: Table 1.** Search masks.** Table 2.** Quality assessment. **Fig. 1.** Prevalences of MIH in different areas of the world.

## Data Availability

Data will be provided on reasonable request to the corresponding author PD Dr. Philipp Sahrmann.
