# Supplementary material for: Is there a rise of prevalence for Molar Incisor Hypomineralization? A meta-analysis of published data
Source: BMC Oral Health. 2024 Jan 25;24:127. doi: 10.1186/s12903-023-03637-0 (PMC10809700; doi:10.1186/s12903-023-03637-0)
Supplement: Supplementary file 1 — Additional file 1: Table 1. Search masks. Table 2. Quality assessment. Fig. 1. Prevalences of MIH in different areas of the world. [file 12903_2023_3637_MOESM1_ESM.docx]

Appendices

**Table 1**

Search masks

**Ovid MEDLINE**

exp Dental Enamel Hypoplasia/ or ((Tooth Discoloration/pa or Dental Enamel/ab or Tooth Abnormalities/ep or Molar/ab) and (hypomineral* or hypo-
mineral* or hypoplas* or opacity*).ti,ab,kw.) or ((molar* or incisor* or tooth or teeth or dental or enamel) adj3 (hypomineral* or hypo-mineral* or hypoplas*
or opacit*)).ti,ab,kw. or (cheese adj3 molar*).ti,ab,kw. or (mottl* adj3 enamel).ti,ab,kw. or (MIH and (tooth or teeth or dent* or orthodont* or orthodent* or
molar*)).ti,ab,kw

AND

Prevalence/ or Cross-Sectional Studies/ or exp "Surveys and Questionnaires"/ or exp Health Surveys/ or (survey or questionnaire or cross-sectional or
prevalence or frequency or population or sample or sampling).ti,ab,kw.

NOT

animals not humans

**Cochrane Library**

((molar* OR incisor* OR tooth OR teeth OR dental OR enamel) NEAR/3 (hypomineral* OR hypo-mineral* OR hypoplas* OR opacit*)):ti,ab,kw
OR (cheese NEAR/3 molar*):ti,ab,kw OR (mottl* NEAR/3 enamel):ti,ab,kw OR (MIH AND (tooth OR teeth OR dent* OR orthodont* OR
orthodent* OR molar*)):ti,ab,kw

AND

(survey OR questionnaire OR cross-sectional OR prevalence OR frequency OR population OR sample OR sampling):ti,ab,kw

**EMBASE**

'molar incisor hypomineralization'/exp OR 'enamel hypoplasia'/exp OR (((molar* OR incisor* OR tooth OR
teeth OR dental OR enamel) NEAR/3 (hypomineral* OR 'hypo mineral*' OR hypoplas* OR opacit*)):ti,ab,kw)
OR ((cheese NEAR/3 molar*):ti,ab,kw) OR ((mottl* NEAR/3 enamel):ti,ab,kw) OR (mih:ti,ab,kw AND
(tooth:ti,ab,kw OR teeth:ti,ab,kw OR dent*:ti,ab,kw OR orthodont*:ti,ab,kw OR orthodent*:ti,ab,kw OR
molar*:ti,ab,kw))

AND

'prevalence'/exp OR 'cross-sectional study'/exp OR 'monitoring'/exp OR 'health survey'/exp OR
'questionnaire'/exp OR survey:ti,ab,kw OR questionnaire:ti,ab,kw OR 'cross sectional':ti,ab,kw OR
prevalence:ti,ab,kw OR frequency:ti,ab,kw OR population:ti,ab,kw OR sample:ti,ab,kw OR sampling:ti,ab,kw

NOT

[animals]/lim OR [humans]/lim) OR [conference abstract]/lim

**Goggle Scholar**

"dental enamel hypoplasia" OR "molar incisor hypoplasia" OR "molar incisor
hypomineralization" OR "molar incisor hypo-mineralization"

AND

survey OR questionnaire OR cross-sectional OR prevalence OR frequency OR
population OR sample OR sampling

**LILACS**

( "DENTAL ENAMEL HYPOPLASIA/" or "dental enamel hypoplasia"
or "molar incisor hypoplasia" or "molar incisor hypomineralization"
or "molar incisor hypo-mineralization" ) or "MOLAR INCISOR
HYPOMINERALIZATION" ) [Words] and ( ( "PREVALENCE/" or
survey or questionnaire or cross-sectional or prevalence or
frequency or population or sample or sampling ) or "SURVEY,
CROSS-SECTIONAL/" ) or "CROSS-SECTIONAL STUDIES/" [Words]

**SCOPUS**

TITLE-ABS-KEY(survey OR questionnaire OR cross-sectional OR prevalence
OR frequency OR population OR sample OR sampling)

**Web of Science**

TS=((molar* OR incisor* OR tooth OR teeth OR dental OR enamel) NEAR/3
(hypomineral* OR hypo-mineral* OR hypoplas* OR opacit*) ) OR TS=(cheese
NEAR/3 molar*) OR TS=(mottl* NEAR/3 enamel) OR TS=(MIH AND (tooth OR
teeth OR dent* OR orthodont* OR orthodent* OR molar*) )
Indexes=SCI-EXPANDED, SSCI, A&HCI, CPCI-S, CPCI-SSH, BKCI-S, BKCI-SSH, ESCI,
CCR-EXPANDED, IC Timespan=All years

AND

TS=(survey OR questionnaire OR cross-sectional OR prevalence OR frequency
OR population OR sample OR sampling)
Indexes=SCI-EXPANDED, SSCI, A&HCI, CPCI-S, CPCI-SSH, BKCI-S, BKCI-SSH, ESCI,
CCR-EXPANDED, IC Timespan=All years

EXCLUDING

WEB OF SCIENCE CATEGORIES: ( ANTHROPOLOGY OR
EVOLUTIONARY BIOLOGY OR ARCHAEOLOGY OR GEOSCIENCES
MULTIDISCIPLINARY OR PALEONTOLOGY OR VETERINARY SCIENCES OR
ZOOLOGY )

**Appendix Table 2** Quality assessment

| **Author year** | **cohort quality** | **sample size** | **Kappa** | | **cohort def.** | **Total** |
| --- | --- | --- | --- | --- | --- | --- |
| Abdalla 2021 | + | + | | + | + | **A** |
| Abdelaziz 2022 | + | + | | 0 | + | **B** |
| Ahmad 2019 | + | + | | + | + | **A** |
| Ahmadi 2012 | + | 0 | | + | + | **B** |
| Al-Hammad 2018 | + | 0 | | + | + | **B** |
| Allazzam 2014 | + | 0 | | + | + | **B** |
| Amend 2021 | + | + | | + | + | **A** |
| Americano 2016 | + | + | | + | + | **A** |
| Arheiam 2021 | + | + | | + | + | **A** |
| Arslanagic 2020 | + | 0 | | + | + | **B** |
| Bahrololoomi 2017 | + | 0 | | 0 | + | **C** |
| Balmer 2005 | + | 0 | | + | + | **B** |
| Balmer 2012 | + | 0 | | + | + | **B** |
| Bhaskar 2014 | + | 0 | | + | + | **B** |
| Biondi 2011 | + | 0 | | + | + | **B** |
| Biondi 2012 | + | 0 | | + | + | **B** |
| Bonzanini 2021 | + | + | | + | + | **A** |
| Buchgrabner 2018 | + | + | | + | + | **A** |
| Calderara 2005 | + | 0 | | + | + | **B** |
| Da Costa Silva 2010 | + | 0 | | + | + | **B** |
| Da Costa Silva 2017 | + | 0 | | + | + | **B** |
| Da Silva 2020 | + | 0 | | + | + | **B** |
| Da Silva J. 2015 | 0 | 0 | | + | + | **C** |
| Dantas-Neta 2016 | + | + | | + | + | **A** |
| Davenport 2019 | + | + | | + | + | **A** |
| Dietrich 2003 | + | 0 | | + | + | **B** |
| Ditto 2018 | + | 0 | | + | + | **B** |
| Dourado 2020 | + | 0 | | + | + | **B** |
| Duarte 2021 | + | + | | + | + | **A** |
| Durmus 2013 | + | 0 | | 0 | + | **C** |
| Einollahi 2020 | + | + | | 0 | + | **B** |
| Elfrink 2012 | + | 0 | | + | + | **B** |
| Elsoud 2019 | + | + | | + | + | **A** |
| Emmatty 2020 | + | 0 | | + | + | **B** |
| Farias 2021 | + | + | | + | + | **A** |
| Farias L. 2021 | + | + | | + | + | **A** |
| Fatturi 2020 | + | + | | + | + | **A** |
| Fernandes 2020 | + | 0 | | + | + | **B** |
| Flexeder 2020 | + | 0 | | 0 | + | **C** |
| Folayan 2018 | + | + | | + | + | **A** |
| Fragelli 2020 | + | + | | + | + | **A** |
| Freitas-Fernandes 2021 | + | + | | + | + | **A** |
| Fteita 2006 | + | 0 | | + | + | **B** |
| Gambetta-Tessini 2018 | + | + | | + | + | **A** |
| Gambetta-Tessini 2019 | + | + | | + | + | **A** |
| García-Margarit 2014 | + | + | | + | + | **A** |
| Georgieva-Dimitrova 2019 | + | 0 | | 0 | + | **C** |
| Ghanim 2012 | + | 0 | | + | + | **B** |
| Ghanim 2014 | + | 0 | | + | + | **B** |
| Glodwanska 2020 | + | + | | 0 | + | **B** |
| Gorbatova 2019 | + | 0 | | 0 | + | **C** |
| Goswami 2019 | + | 0 | | 0 | + | **C** |
| Groselj 2013 | + | 0 | | + | + | **B** |
| Gurrusquieta 2017 | + | 0 | | + | + | **B** |
| Gutierrez 2019 | + | + | | + | + | **A** |
| Hamdan 2020 | + | + | | + | + | **A** |
| Hanan 2015 | + | 0 | | + | + | **B** |
| Hartsock 2020 | + | 0 | | + | + | **B** |
| Hernandez 2018 | + | + | | + | + | **A** |
| Hertel 2017 | + | 0 | | 0 | + | **C** |
| Hong 2017 | + | 0 | | 0 | + | **C** |
| Hoyte 2020 | + | 0 | | 0 | + | **C** |
| Hussain 2018 | + | + | | + | + | **A** |
| Hussein 2019 | + | 0 | | + | + | **B** |
| Hysi 2016 | + | + | | + | + | **A** |
| Irigoyen-Camacho 2020 | + | + | | + | + | **A** |
| Jalevik 2001 | + | 0 | | 0 | + | **C** |
| Jalevik 2018 | + | 0 | | 0 | + | **C** |
| Jankovic 2014 | + | 0 | | + | + | **B** |
| Jasulaityte 2007 | + | 0 | | + | + | **B** |
| Jasulaityte 2008 | + | 0 | | + | + | **B** |
| Jeremias 2013 | + | + | | + | + | **A** |
| Jurlina 2020 | + | + | | + | + | **A** |
| Kemoli 2008 | + | 0 | | 0 | + | **C** |
| Kevrekidou 2015 | + | + | | + | + | **A** |
| Kilinc 2019 | + | 0 | | 0 | + | **C** |
| Kim 2016 | + | 0 | | + | + | **B** |
| Kirthiga 2015 | + | 0 | | + | + | **B** |
| Koruyucu 2018 | + | + | | + | + | **A** |
| Krishnan 2015 | + | 0 | | + | + | **B** |
| Kühnisch 2014 | + | 0 | | + | + | **B** |
| Kühnisch 2018 | + | 0 | | + | + | **B** |
| Kukleva 2008 | + | 0 | | 0 | + | **C** |
| Kuscu 2008 | + | 0 | | + | + | **B** |
| Kuscu 2009 | + | 0 | | + | + | **B** |
| Li 2012 | + | 0 | | + | + | **B** |
| Liena 2020 | + | 0 | | 0 | + | **C** |
| Lopez 2014 | + | 0 | | + | + | **B** |
| Lygidakis 2008 | + | 0 | | + | + | **B** |
| Mahima 2020 | + | + | | + | + | **A** |
| Mahoney 2009 | + | 0 | | + | + | **B** |
| Mahoney 2011 | + | 0 | | 0 | + | **C** |
| Martinez-Gomez 2011 | + | 0 | | 0 | + | **C** |
| Martinovic 2017 | + | 0 | | + | + | **B** |
| Mejia 2019 | + | + | | + | + | **A** |
| Menoncin 2019 | + | + | | + | + | **A** |
| Mishra 2016 | + | 0 | | + | + | **B** |
| Mittal 2014 | + | + | | + | + | **A** |
| Mittal 2016 | + | + | | + | + | **A** |
| Mittal 2016/2 | + | 0 | | + | + | **B** |
| Mulic 2017 | + | 0 | | + | + | **B** |
| Munoz 2011 | + | 0 | | 0 | + | **C** |
| Muratbegovic 2008 | + | + | | + | + | **A** |
| Murrieta-Pruneda 2016 | + | 0 | | + | + | **B** |
| Negre-Barber 2018 | + | + | | + | + | **A** |
| Ng 2015 | + | 0 | | 0 | + | **C** |
| Nisii 2022 | + | + | | + | + | **A** |
| Nsour 2018 | + | + | | + | + | **A** |
| Ofi 2015 | + | + | | 0 | + | **B** |
| Ordonez 2019 | + | + | | + | + | **A** |
| Orellana-Herrera 2020 | + | + | | + | + | **A** |
| Oyedele 2015 | + | + | | + | + | **A** |
| Oyedele 2015/2 | + | + | | + | + | **A** |
| Petrou 2014 | + | 0 | | + | + | **B** |
| Pitiphat 2014 | + | 0 | | + | + | **B** |
| Pitiphat 2014/2 | + | 0 | | + | + | **B** |
| Portella 2019 | + | + | | + | + | **A** |
| Poureslami 2018 | + | + | | 0 | + | **B** |
| Preusser 2007 | + | 0 | | 0 | + | **C** |
| Quispe 2022 | + | + | | + | + | **A** |
| Rai 2018 | + | + | | + | + | **A** |
| Rai 2019 | + | + | | + | + | **A** |
| Raposo 2019 | + | + | | + | + | **A** |
| Ray 2020 | + | 0 | | + | + | **B** |
| Reis 2021 | + | 0 | | + | + | **B** |
| Rizk 2018 | + | 0 | | + | + | **B** |
| Rodriguez-Rodriguet 2021 | + | + | | + | + | **A** |
| Saber 2018 | + | 0 | | 0 | + | **C** |
| Saitoh 2018 | + | 0 | | 0 | + | **C** |
| Sakly 2018 | + | 0 | | + | + | **B** |
| Salem 2016 | + | 0 | | + | + | **B** |
| Salih 2012 | + | 0 | | 0 | + | **C** |
| Santos 2019 | + | + | | + | + | **A** |
| Schmalfuss 2016 | + | 0 | | + | + | **B** |
| Shin 2010 | + | 0 | | + | + | **B** |
| Shin 2017 | + | 0 | | + | + | **B** |
| Shojaeepour 2020 | + | 0 | | 0 | + | **C** |
| Shrestha.R 2014 | + | 0 | | + | + | **B** |
| Sidaly 2017 | + | 0 | | + | + | **B** |
| Singh 2020 | + | 0 | | 0 | + | **C** |
| Sönmez 2013 | + | 0 | | + | + | **B** |
| Sosa-Soto 2021 | + | + | | + | + | **A** |
| Subramaniam 2016 | + | + | | + | + | **A** |
| Tadikonda 2015 | + | + | | + | + | **A** |
| Tagelsir 2020 | + | 0 | | + | + | **B** |
| Tarannum 2021 | + | 0 | | 0 | + | **C** |
| Temilola 2015 | + | + | | + | + | **A** |
| Temilola 2015/2 | + | + | | + | + | **A** |
| Thakur 2020 | + | 0 | | 0 | + | **C** |
| Tourino 2016 | + | + | | + | + | **A** |
| Verma 2022 | + | 0 | | 0 | + | **C** |
| Villanueva-Gutiérrez 2019 | + | + | | + | + | **A** |
| Villanueva-Gutiérrez 2019/2 | + | + | | + | + | **A** |
| Wuollet 2016 | + | 0 | | + | + | **B** |
| Wuollet 2018 | + | 0 | | + | + | **B** |
| Yannam 2016 | + | 0 | | 0 | + | **C** |
| Yi 2021 | + | 0 | | + | + | **B** |
| Zakirulla 2018 | 0 | 0 | | 0 | + | **C** |
| Zawaideh 2011 | + | 0 | | + | + | **B** |

+ – high quality standards reported in the study 0 – no respective data for quality assessment reported or reporting low quality standards

A – high overall quality B – moderate overall quality C - low overall quality

**Appendix Fig. 1. Prevalences of MIH in different areas of the world**


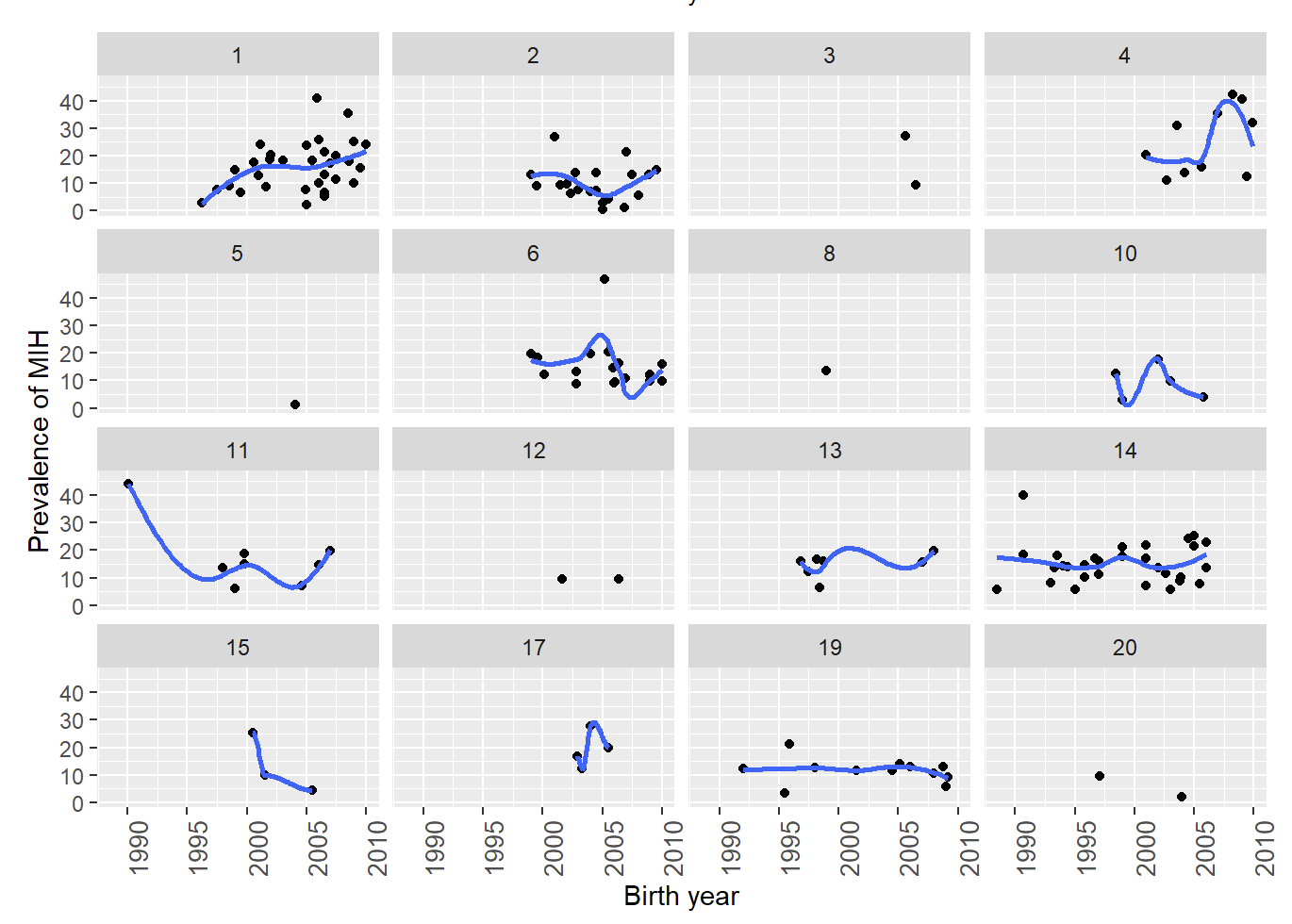


Reported prevalences of MIH related to birthyear of the respective cohort.

1 - North African and Middle East, 2 - South Asia, 3 - Andean Latin America, 4 - Central Latin America, 5- Latin America and Caribbean, 6 - Tropical Latin America, 7 - Central Sub-Saharan Africa, 8-Eastern Sub-Saharan Africa, 9 - Southern Sub-Saharan Africa, 10 - Western Sub-Saharan Africa, 11 - High-income Asia Pacific, 12 - High-income North America, 13 - Southern Latin America, 14 - Western Europe, 15 - East Asia, 16 - Oceania 16, 17 - Southeast Asia, 18 - Central Asia, 19 - Central Europe 19, 10 - Eastern Europe

Each black dot indicates the prevalence reported in an individual study. The blue line shows the loess-smoothed prevalence of all studies related to publication year or birth year, while the grey area indicates 95% confidence interval
